# Effect of hydroalcoholic extract of *Berberis integerrima* and resveratrol on ovarian morphology and biochemical parameters in Letrozole-induced polycystic ovary syndrome rat model: An experimental study

**DOI:** 10.18502/ijrm.v13i8.7505

**Published:** 2020-08-19

**Authors:** Fatemeh Ashkar, Mohammad Hassan Eftekhari, Nader Tanideh, Farhad Koohpeyma, Maral Mokhtari, Cambyz Irajie, Aida Iraji

**Affiliations:** ^1^Department of Clinical Nutrition, School of Nutrition and Food Sciences, Shiraz University of Medical Sciences, Shiraz, Iran.; ^2^Stem Cells Technology Research Center, Shiraz University of Medical Sciences, Shiraz, Iran.; ^3^Department of Endocrinology, Endocrinology and Metabolism Research Center, Shiraz University of Medical Sciences, Shiraz, Iran.; ^4^Department of Pathology, School of Medicine, Shiraz University of Medical Sciences, Shiraz, Iran.; ^5^Department of Medical Biotechnology, School of Advanced Medical Sciences and Technologies, Shiraz University of Medical Sciences, Shiraz, Iran.; ^6^Medicinal and Natural Products Chemistry Research Center, Shiraz University of Medical Sciences, Shiraz, Iran.

**Keywords:** Barberry, Resveratrol, Polycystic ovary syndrome, Ovary, Rat.

## Abstract

**Background:**

Resveratrol and *Berberis integerrima *(*B. integerrima*) are known to be natural antioxidants and regulators of human metabolism. However, the effects of resveratrol and *B. integerrima* on the ovarian morphology in polycystic ovary syndrome (PCOS) are not obvious.

**Objective:**

This study aimed to determine the effect of the hydroalcoholic extract of *B. integerrima* in combination with resveratrol on some biochemical parameters and ovarian morphology in the letrozole-induced PCOS rat.

**Materials and Methods:**

Seventy adult female Sprague-Dawley rats aged 10-12 weeks weighing 200 ± 20 gr were randomly divided into seven groups (n = 10/each). Group I): normal; Group II): vehicle; Group III): letrozole-induced PCOS 1 mg/kg letrozole orally, rats receiving 1 cc normal saline orally; Group IV): PCOS + receiving 150 mg/kg metformin orally; Group V): PCOS + receiving 20 mg/kg resveratrol orally; Group VI): PCOS + 3 gr/kg barberry orally; and Group VII): PCOS + receiving 3 gr/kg barberry and 20 mg/kg resveratrol orally. All animals were followed-up for 63 days. The biochemical parameters and histological assessments of ovaries were performed.

**Results:**

Resveratrol alone and/or in combination with *B. integerrima* treatment in rats led to a significant decrease in low-density lipoprotein, triglyceride, malondialdehyde, and tumor necrosis factor-alpha concentrations (p = 0.02). The groups IV, V, VI, and VII showed a decrease in insulin resistance and an increase in the superoxide dismutase, total antioxidant capacity, and high-density lipoprotein (p = 0.01). No significant difference was observed between the level of serum glucose in the treatment groups. Number of cystic follicles had a significant decrease in barberry, resveratrol, and their combination groups (p < 0.001).

**Conclusion:**

Resveratrol, *B. integerrima*, and their combination as natural products with fewer side effects might be effective as an alternative medicine in treatment of PCOS.

## 1. Introduction

Polycystic ovary syndrome (PCOS) is one of the most common endocrine disorders of fertility in women that affects 6-14% of them during the reproductive years. In the Iranian population, the prevalence of PCOS has been reported to be 7.1%, 11.7%, and 14.6% according to the National Institute of Health, Androgen Excess Society criteria, and the Rotterdam consensus, respectively (1, 2).

This syndrome is associated with a range of reproductive, endocrine, and metabolic specifications, including obesity, being overweight, hyperandrogenism, anovulation, and infertility that results in hyperinsulinism and type II diabetes.

There are numerous researches about inflammation in chronic diseases such as PCOS, and the inflammatory markers include C-reactive (CRP) protein, interleukin (IL) 6, and tumor necrosis factor alpha (TNF-α). As a result of inflammation, the risk of developing type 2 diabetes, cardiovascular disease, ovarian dysfunction, and hyperandrogenism increases because of the free radicals. Furthermore, oxidative damage is aggravated by the decrease in the body's antioxidant defense mechanisms such as superoxide dismutase (SOD) and catalase (CAT) activities that act as free radicals' scavengers. Total antioxidant capacity (TAC) is sensitive to changes in plasma antioxidant levels and degree of insulin resistance. Therefore, a wide range of oxidative stress biomarkers, including malondialdehyde (MDA), protein carbonyl, TAC, SOD, glutathione peroxidase (GPx), and glutathione (GSH) have been tested in the patients (2-4).

Due to the low efficacy and safety of the synthetic drugs and the increased risk of insulin resistance and hyperlipidemia, there is a growing tendency toward the consumption of medicinal plants instead of synthetic compounds (5).

*B. integerrima* is a regular ingredient of Iranian cuisine (6). The high antioxidant capacity of this plant is because of the presence of natural flavonoids and phenolic compounds such as anthocyanins and carotenoids in *Berberis* which are known to be antidiabetic, anticancer, and antimicrobial natural agents. Research on the cytotoxicity of *B. integerrima* extract demonstrated no cellular toxicity (7-9).

Resveratrol (3, 5, 4'-trihydroxy-trans-stilbene) is commonly found in grapes, peanuts, and some types of berries as a natural polyphenolic compound. It is recognized as a powerful antioxidant with antiplatelet, anticancer, anti-inflammation, and antiaging effects. Several studies have demonstrated direct antioxidant effects of resveratrol, and its protective effects may be due to the enhancement of antioxidant capacity in both humans and rodents (10, 11). As a result, antioxidants are extremely important because they inactivate the reactive species and increase the protection of biological sites (9).

In this study, the effectiveness of resveratrol, *B. integerrima*, and their combination were evaluated in the PCOS rat models for the first time. Moreover, the biochemical parameters, including oxidative stress, inflammatory and lipid profiles, as well as ovarian pathology were determined.

## 2. Materials and Methods

### Induction of PCOS

**Figure 1:**
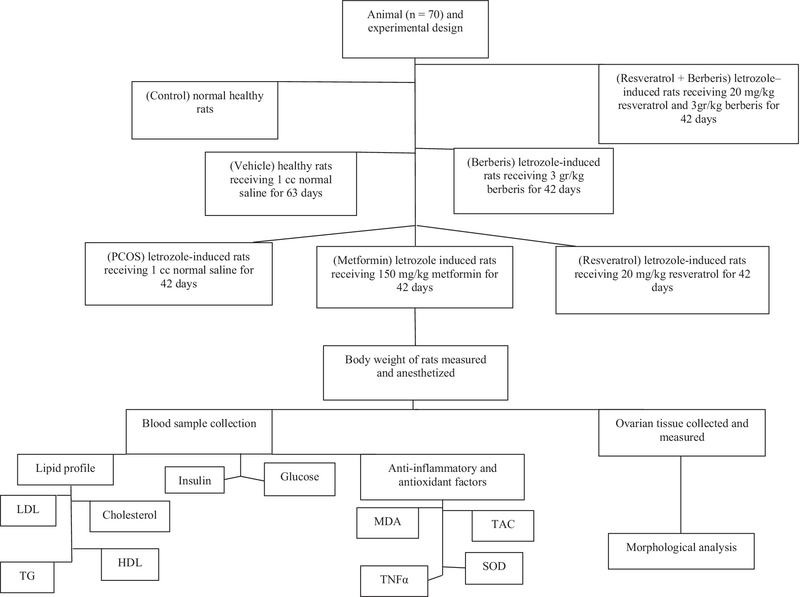
Flow chart of the experimental design. PCOS Polycystic ovary syndrome; MDA, Malondialdehyde; TAC: Total antioxidant capacity; SOD: Superoxide dismutase; TNF-α: Tumor necrosis factor alpha; LDL: Low-density lipoprotein; HDL: High-density lipoprotein; TG: Triglyceride.

Seventy adult female Sprague-Dawley rats aged 10-12 wk weighing 200 ± 20 gr were used in this experimental study. The rats were kept ad libitum in steel cages with controlled temperature and humidity, 22 ± 2-C and 55 ± 5%, respectively, at 12 hr light/dark cycle. Letrozole orally induced PCOS in rats; this model has histological and biochemical similarities with PCOS in humans. This was done by inducing 1 mg/kg letrozole (Aburaihan Pharma.co., Tehran, Iran)dissolved in 0.9% NaCl solution (Razi Pharma.co., Tehran, Iran) administered orally for 21 days (12). The ﬂowchart of the study is shown in Figure 1.

### Experimental design

The rats were randomly divided into seven groups (n = 10/ each) as follows:

• Group (I): control, healthy rats not receiving any interventions;

• Group (II): vehicle, healthy rats receiving 1 cc normal saline for 63 days;

• Group (III): letrozole-induced PCOS (Aburaihan Pharma.co., Tehran, Iran), rats receiving 1 cc normal saline for 42 days orally;

• Group (IV): PCOS + receiving 150 mg/kg metformin (Shafa Pharma.co., Tehran, Iran) dissolved in 1 cc normal saline for 42 days orally, after letrozole-induced PCOS (13);

• Group (V): PCOS + receiving 20 mg/kg resveratrol (Nutrivit Co., USA) dissolved in 1 cc normal saline for 42 days orally, after letrozole-induced PCOS (11);

• Group (VI): PCOS + 3 gr/kg barberry dissolved in 1 cc normal saline for 42 days orally, after letrozole-induced PCOS (14);

• Group (VII): PCOS + receiving 3 gr/kg barberry and 20 mg/kg resveratrol dissolved in 1 cc normal saline for 42 days orally, after letrozole-induced PCOS.

All animals were weighed on day 1, after PCOS induction (day 21), and at the end of the study (day 63). After 63 days, all study groups were made to fast overnight and were anesthetized via ketamine (Alfasan.co., Holland) (100 mg/kg) and xylazine (Alfasan.co., Holland) (10 mg/kg). Blood samples were collected by cardiocentesis, and their sera were separated to be used for the assessment of biochemical parameters. Afterward, the animals were sacrificed and their ovaries were harvested, cleaned of fat, weighed, and fixed in 10% formalin.

### Barberry extract preparation

To prepare the extract, powdered fruit of *B. integerrima* (herbarium number 2518) was mixed with 70% ethanol (Razi Pharma.co., Tehran, Iran), stirred for 72 hr, and placed in a percolator. The remainders were placed in rotary devices and dried in a vacuum in color glass until usage.

### Antioxidant activity

2, 2-diphenyl-1-picrylhy-drazyl (DPPH) assay is a powerful technique to determine the scavenging potential of antioxidant. DPPH is characterized as a stable radical containing the delocalization electron with deep violet color (15). The odd electron of DPPH absorbed a hydrogen atom from antioxidants and the reduction of violet color took place. The antioxidant capacity was calculated by measuring the reduction in the absorbance at 517 nm. The modified Brand-Williams method has been used in this study (16). Plants extract was diluted with methanol at various concentrations. Quercetin as a positive control was used. A stock solution of DPPH (110 μM) was prepared freshly by dissolving the appropriate amount in methanol. A volume of 20 μl of different concentrations of the sample extract or standard liquid and 180 μl of methanol (with or without DPPH) were mixed in 96-well plate at 25°C temperature for 30 min (17).

The DPPH scavenging effect was calculated as follows:

Scavenging effect (%) = 

$$\frac{A0-A1}{A0}\times 100$$

Where A${}_{0}$ is the absorbance of the control, and A${}_{1}$ is the absorbance in the presence of the sample. All determinations were performed in triplicate.

### Determination of total phenolic content (TPC) by Folin-Ciocalteu assay

Based on the Folin-Ciocalteu method, 10 μL of Folin-Ciocalteu reagent and 5 μL of the tested sample or gallic acid as standard were added to 96-well plate and diluted with 160 μL distilled water. The contents were then mixed thoroughly for around 10 min. After 10 min of incubation, 30 μL of 25% Na${}_{2}$CO${}_{3}$ was added and the mixture was further incubated for 90 min at room temperature in the dark. Finally, the absorbance was measured at 725 nm (18) and the concentration of the total phenolic was calculated as milligrams of gallic acid equivalents per gram of dry extract.

### Biochemical markers

Fasting blood glucose (FBG) was measured by an enzymatic colorimetric method using a biochemical Auto-analyzer (BT-1500, Italy) and a commercial diagnostic kit (Pars Azmoon Co., Iran).

Lipid profiles [total cholesterol (TC), triglyceride (TG), low-density lipoprotein (LDL), and high- density lipoprotein (HDL)] were estimated via the enzymatic colorimetric method using a biochemical Auto-analyzer (BT-1500, Italy) and the kits (Pars Azmoon Co., Iran).

SOD and TAC were determined via the enzymatic colorimetric method with the microplate reader using enzymatic kits (ZV-TAC-A96) purchased from Zellbio Co., Germany. Additionally, MDA activity was determined via spectrophotometric analysis using thiobarbituric acid reactive substances (TBARS) method (19).

Plasma insulin level was measured via enzyme-linked immunosorbent assay (ELISA) method using insulin kits (Rat Insulin Mercodia 10-1250-01, Sweden) (2).

The homeostasis model assessment of insulin resistance (HOMA-IR) was calculated using the following formula (20): 

 Fasting  plasma  insulin μUmL× fasting  plasma  glucose  mM 22.5

TNF-α was measured by the ELISA method using TNF-α kits (865.000.96 Diaclone, France) (21).

### Ovarian histopathology 

Consecutive serial sections with 5-micron thickness from formalin-fix ovary were prepared, and then from each 10 sections one slide and from each ovarian sample 3 sections were stained with hematoxylin and eosin (22). The thin sections were dehydrated, cleared, and eventually mounted within EntellanⓇ (Merck Co., Germany) and cover slipped. The prepared slides were evaluated using a light microscope (Olympus CX31, Japan).

### Ethical consideration

The study was conducted in accordance with the recommendations of the European Council Directive (86/609/EEC) on November 24, 1986, regarding the protection of animals used for experimental purposes (http://data.europa.eu/eli/dir/1986/609/oj). The animal protocols were also approved by the Ethics Committee of Shiraz University of Medical Sciences, Shiraz, Iran (Code: IR. SUMS. REC.1394.S1208).

### Statistical analysis

The results have been reported as mean ± standard deviation. Additionally, data were analyzed using one-way analysis of variance (ANOVA) followed by Tukey's test. P < 0.05 was considered to be statistically significant. All analyses were done using the SPSS statistical software, version 22 (SPSS Inc., Chicago, IL, USA).

## 3. Results 

###  Biochemical assessment 

According to Table I, the PCOS-induced group caused significant changes in serum lipid levels compared to the controls. Additionally, TG, TC, and LDL levels meaningfully increased (p = 0.02, p = 0.01, and p = 0.01, respectively), while the HDL level decreased significantly (p = 0.01) compared to the controls. However, metformin, barberry, and resveratrol significantly reduced TG (p = 0.01), TC (p = 0.01), and LDL (p = 0.01) levels compared to the group with PCOS. Nevertheless, the results showed no significant changes between the barberry + resveratrol rats and resveratrol, and barberry groups related to the levels of HDL, LDL, TC, and TG (p ≤ 0.05). There was no significant difference between the treatment groups.

**Figure 2:**
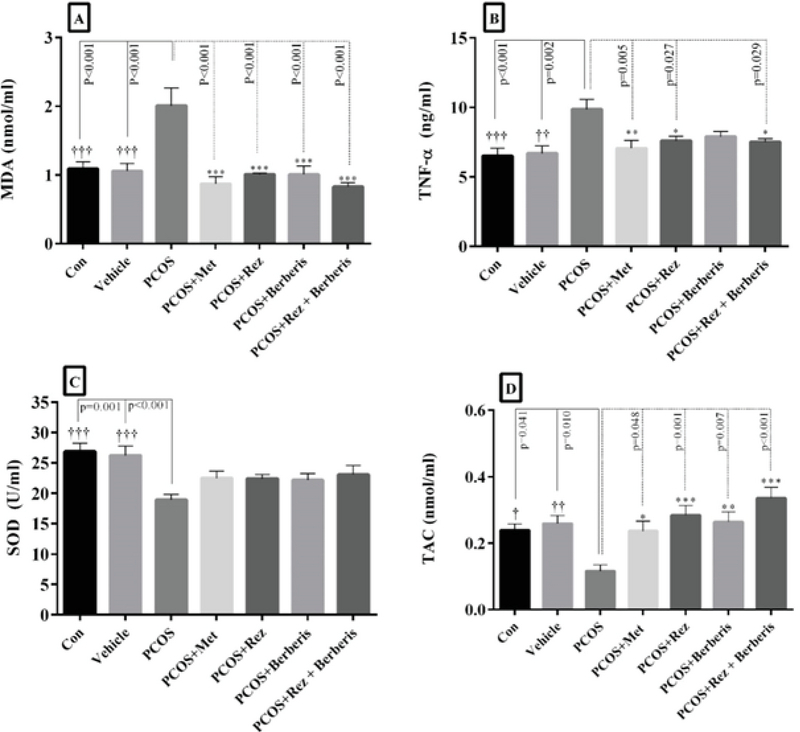
The measurement of antioxidant activities and inflammatory factor in different groups. PCOS: Polycystic ovary syndrome; Met: Metformin; Rez: Resveratrol; MDA: Malondialdehyde; TNF-α: Tumor necrosis factor-α; SOD: Superoxide dismutase; TAC: Total antioxidant capacity †p < 0.05, PCOS vs control and vehicle groups; *p < 0.05, PCOS vs PCOS + Met, PCOS + Rez, PCOS + berberis, and PCOS + Rez + berberis groups *p < 0.05, †p < 0.05, **p < 0.01, ††p < 0.01, ***p < 0.001, †††p < 0.001

The effects of barberry and resveratrol on antioxidant activity and inflammatory factors are presented in Figure 2. Accordingly, the PCOS-induced group (without treatment) showed a significant decrease in SOD (p = 0.01) and TAC (p = 0.04) levels, but an increase in TNFα (p = 0.01) and MDA (p = 0.01) concentrations. Treatment with metformin, resveratrol, and barberry could restore SOD (p = 0.01) and TAC (p = 0.01) levels close to those in the control groups. However, MDA (p = 0.01) and TNF-α (p = 0.02) levels reduced significantly in metformin-, resveratrol-, and barberry-treated groups in comparison to the control groups. Nonetheless, no significant differences between the barberry + resveratrol rats and those in the resveratrol and barberry groups related to MDA, SOD, TNF-α, and TAC concentrations (p = 0.99) were observed. There was no significant difference between the treatment groups.

Table II shows the ovarian weights of rats in the control and experimental groups. The ovarian weight increased significantly in the PCOS rats in comparison to the control rats (p = 0.01). Treatment with metformin, barberry, resveratrol, and barberry + resveratrol significantly decreased the ovarian weight in PCOS rats after 42 days (p = 0.01). The ovarian weights were not significant between the barberry + resveratrol rats and those in the resveratrol and barberry groups (p = 0.99).

### Insulin resistance

According to the results presented in Table II, the insulin level and HOMA-IR score significant changes in the metformin-, resveratrol-, and barberry-treated groups compared to the control groups (p = 0.007, p = 0.004, respectively) demonstrated significant differences. The results also demonstrated no significant differences between the barberry + resveratrol rats and those in resveratrol and barberry groups with respect to insulin level, glucose level, and HOMA-IR score (p = 0.99).

### Histomorphological changes

**Figure 3:**
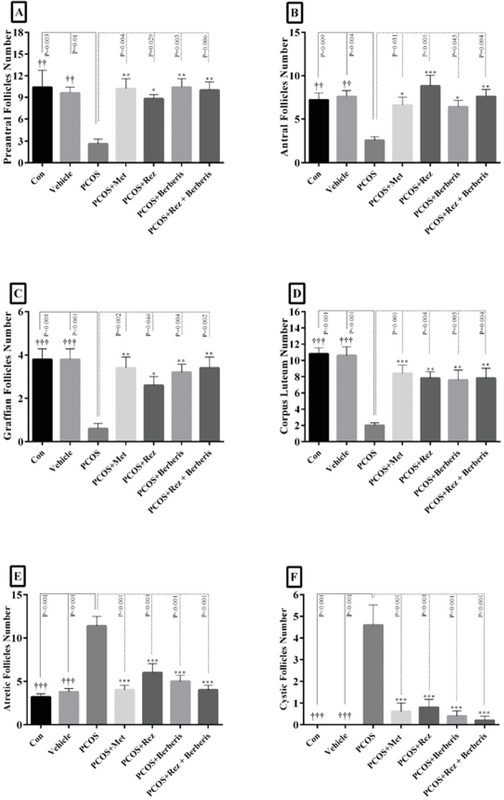
Presentation of the follicle numbers in different groups. PCOS: Polycystic ovary syndrome; Met: Metformin; Rez: Resveratrol A: Preantral follicles; B: Antral follicles; C: Graffian follicles; D: Corpus luteum; E: Atrelic follicles; F: Cystic follicles †p < 0.05, PCOS vs control and vehicle groups; *p < 0.05, PCOS vs PCOS + Met, PCOS + Rez, PCOS + berberis, and PCOS + Rez + berberis groups, *p < 0.05, †p < 0.05, **p < 0.01, ††p < 0.01, ***p < 0.001, †††p < 0.001

**Figure 4:**
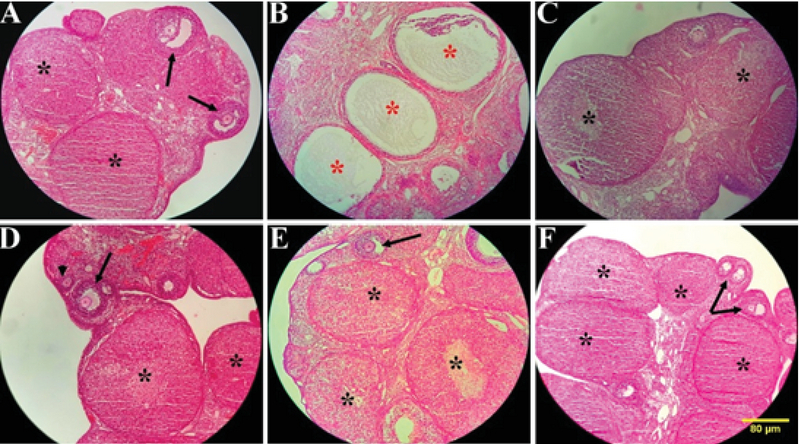
Images of ovaries in different stages of the intervention. (Magnification ×100). (A) Control group. (B) PCOs group. (C) PCOS + metformin group. (D) PCOS + resveratrol group. (E) PCOS + barberry group. (F) PCOS + resveratrol + barberry group. The arrows in A, D, E, and F indicate normal antral follicles. The red stars in B indicate ovarian cysts. The black stars in A, C, D, E, and F indicate corpus luteum, and pick arrow in D indicate preantral follicles.

In this study, a significant decrease in the number of preantral follicles, antral follicles, graafian follicles, and corpus luteum in PCOS group compared to the control and vehicle (p = 0.01) groups is observed (Figure 3A-D). On the other hand, the number of follicles increased significantly in comparison with PCOS (p = 0.01). The number of atretic and cystic follicles increased significantly in the PCOS group in contrast with the control and vehicle groups; while, on the other hand, they decreased significantly in barberry, resveratrol and their combination groups (p = 0.01) (Figure 3E-F). Ovarian sections of control group animals exhibited healthy follicles with oocytes at different development stages (Figure 4A). PCOS- induced group exhibited numerous subcapsular cysts with very thin or no granulosa layers. Additionally, complete absence of corpora lutea were noticed, representing anovulation. Few follicles were observed at their early stages of development. In addition, they were accompanied by atretic follicles containing fluid-filled antrum and a larger number of pyknotic granulosa cells (Figure 4B). On the other hand, metformin treatment group led to the removal of the cysts and appearance of healthy follicles and corpora lutea (Figure 4C). Sections of the resveratrol (20 mg/kg) group exhibited larger follicles and few corpora lutea. Indeed, the cysts were absent and normal-sized healthy follicles were found at different development stages (Figure 4D). Photomicrograph from the barberry group revealed an increase in this group's corpus luteum (Figure 4E). Treatment with barberry and resveratrol also resulted in the appearance of many corpora lutea and antral follicles with clearly differentiated oocytes, granulosa cell layers, corona radiates, cumulus oophorus, and theca cells (Figure 4F).

### Determination of TPC and antioxidant activity

The DPPH antioxidant assay was performed to determine the ability of the sample to inactivate DPPH radical. The IC${}_{50}$ of Berberis hydroalcholic extract and Quercetin as positive control were 33.1 ± 1.07 μg/mL and 9.43 ± 2.26 μM, respectively.

The TPC of the extract as determined by Folin-Ciocalteu method are reported as gallic acid equivalent. As expected, the amount of total phenolic compounds was found to be rich in Berberis hydroalcoholic extract (214.12 ± 6.95 mg GAE/gr dry extract). Values were expressed as mean ± standard error of mean (SEM) of triplicate experiments.

**Table 1:** Measurements of lipid profiles in different groups


**Groups**	**LDL (mg/dl)**	**HDL (mg/dl)**	**TG (mg/dl)**	**Cholesterol (mg/dl)**
**Control**	83.35 ± 3.43	45.40 ± 2.96	78.86 ± 4.66	115.2 ± 2.67
**Vehicle**	85.26 ± 4.10	44.20 ± 1.93	81.22 ± 7.09	114.3 ± 2.77
**PCOS**	114.7 ± 4.15†	31.50 ± 2.00†	111.43 ± 8.04†	131.6 ± 3.11†
**PCOS + Met**	91.80 ± 2.74${}^{*}$	38.10 ± 2.66	75.56 ± 7.31${}^{*}$	111.2 ± 4.44${}^{*}$
**PCOS + Rez**	93.25 ± 2.87${}^{*}$	41.90 ± 2.30	81.50 ± 3.67${}^{*}$	118.6 ± 2.49
**PCOS + berberis**	93.57 ± 1.85${}^{*}$	36.40 ± 2.39	78.78 ± 6.42${}^{*}$	113.8 ± 2.60${}^{*}$
**PCOS + Rez + berberis**	92.64 ± 1.75${}^{*}$	44.40 ± 3.06${}^{*}$	73.89 ± 5.71${}^{*}$	114.6 ± 3.98${}^{*}$
PCOS: Polycystic ovary syndrome; Met: Metformin; Rez: Resveratrol; LDL: Low-density lipoprotein; HDL: High density lipoprotein; TG: Triglyceride, Data presented as Mean ± SD †p < 0.05, PCOS vs. control and vehicle groups; *P < 0.05, PCOS group vs. PCOS + Met, PCOS + Rez				

**Table 2:** Measurements of insulin and glucose concentrations, HOMA-IR score, and ovarian weight in different groups


**Groups**	**Ovarian weight (mg)**	**Insulin (µu/ml)**	**Glucose (mmol/L)**	**HOMA-IR**
**Control**	0.021 ± 0.001	9.85 ± 1.71	5.89 ± 0.49	2.58 ± 0.38
**Vehicle**	0.020 ± 0.001	8.78 ± 2.16	6.36 ± 0.36	2.48 ± 0.63
**PCOS**	0.045 ± 0.002†	20.47 ± 4.58†	6.49 ± 0.41	5.90 ± 1.49†
**PCOS + Met**	0.024 ± 0.002${}^{*}$	10.41 ± 1.43${}^{*}$	6.13 ± 0.68	2.84 ± 0.72${}^{*}$
**PCOS + Rez**	0.028 ± 0.001${}^{*}$	10.57 ± 1.76${}^{*}$	6.69 ± 0.34	3.15 ± 1.08${}^{*}$
**PCOS + berberis**	0.029 ± 0.002${}^{*}$	8.68 ± 1.53${}^{*}$	6.10 ± 0.32	2.36 ± 0.23${}^{*}$
**PCOS + Rez + berberis**	0.026 ± 0.001${}^{*}$	9.47 ± 1.78${}^{*}$	5.94 ± 0.26	2.50 ± 0.88${}^{*}$
Data presented as Mean ± SD PCOS: Polycystic ovary syndrome; Met: Metformin; Rez: Resveratrol Homa-IR: Homeostatic model assessment for insulin resistance †p < 0.05, PCOS vs. control and vehicle groups; *p < 0.05 PCOS group vs. PCOS + Met, PCOS + Rez, PCOS + berberis, and PCOS + Rez + berberis groups				

## 4. Discussion

Generally, the animal models are used to gain clinical insight and high degree of evolutionary conservation into human reproductive disorders, especially PCOS (23). PCOS induced by letrozole is histologically and biochemically similar to PCOS in humans that increases testosterone but decreases progesterone and estrogen levels, eventually leading to the appearance of cysts that inhibit the aromatase enzyme (24). Based on Webber and colleagues study in 2003, PCOS could result in an increase in the number of antral follicles, ovarian stroma, hyperplasia theca cells, and ovarian cortical thickness (25). Moreover, it can be concluded that the number of primordial and primary forms of prenatal follicles is increased in PCOS compared with normal ovaries. Indeed, the number of primary follicles (early growing) is increased significantly in both ovulatory and anovulatory PCOS, with a reciprocal decrease in the proportion of primordial follicles compared to normal ovaries. These results are in line with those of the present investigation.

The histomorphological slides in the resveratrol group showed fewer cysts and the presence of corpus luteum in the ovaries, indicating follicle maturation and ovulation. This was in the same line with the results of a previous study by Ergenoglu and co-workers In that study, the treatment of PCOS-induced rats with 10 mg/kg resveratrol led to the development of antral follicles to the normal state (11). These favorable changes indicate that barberry, resveratrol, and their combination may have positive effects on PCOS symptoms. Interestingly, the histomorphological changes in the barberry, resveratrol, and their combination groups were totally comparable to those in the metformin-treated group.

In this study, it was found that *B. integerrima* is a predominant source of antioxidant (DPPH, assay, IC${}_{50}$ = 33.1 + 1.07 µg/mL). Our data are supported by a few investigations on antioxidant activity and phenolic content amounts of Berberis species (26-28). High amounts of antioxidant and polyphenol present in Berberis act as free radical scavengers, making it a necessary aid to human health.

Our results revealed a significant increase in body weight among the rats suffering from letrozole-induced PCOS in comparison with the control and vehicle groups, which was consistent with the results obtained by Maharjan (29). On the other hand, our findings confirmed that there is a significant decrease in body weight in the groups treated with barberry, metformin, and resveratrol. These effects are consistent with the previously published data.

Evidence indicate that there is an excessive occurrence of alterations in lipid profiles and lipedema in women with PCOS (30). The current results demonstrated that barberry extract and resveratrol could reduce TC, LDL, and TG levels following an increase in HDL concentrations. Besides, the simultaneous use of barberry and resveratrol provides better results in comparison with metformin in terms of HDL and TG concentration.

Recent studies have shown that Peroxisome proliferation-activated receptor-α (PPAR-α) is predominantly expressed in tissues that have fatty acids metabolizing function such as liver, heart, kidney, and muscles. Activation of PPAR-α could reduce serum TG and also raise HDL level. Barberry is able to activate PPAR-α and AMP-activated protein kinase (AMPK) which is accompanied by reduction of lipid accumulation in adipocytes (31). Similarly, Derosa and colleagues examined the potential application of Berberine in the regulation of plasma lipids in patients with cardiovascular diseases and found that Berberine present in barberry could decrease TC, LDL, and TG levels following an increase in HDL concentration (32).

Another study found that Berberine might improve the liver function and secretion of bile acids. It seems that the high levels of polyphenolic compounds might inhibit intestinal cholesterol absorption, inactivate 3-hydroxy-3-methyl-glutaryl-CoA reductase (HMGCoA) enzyme, and reduce the intestinal production of chylomicrons in berberine which in turn results in constructive effects of this substance on lipid profiles (33).

The hypocholesterolemia-inducing effect of resveratrol may be explained by its phenolic hydroxyls compounds that result in oxidation of unsaturated fatty acids, as well as a decrease in circulating cholesterol, downregulation of HMG-CoA reductase, and an increase in the expression of the LDL receptors (LDL-R) in hepatocytes. In addition, it is reported that the alternations in medium and long-chain triglyceride concentrations, decreased apolipoprotein C-III, reduced activity of hepatic acyl-CoA cholesterol acyltransferase, and the upregulation of genes involved in lipid metabolism might be linked to the hypotriglyceridemic effect of resveratrol which is in line with our findings (34-36).

A clinical trial demonstrated that 250-1000 mg daily consumption of resveratrol declined LDL in patients with type II diabetes. In the same way, treatment with resveratrol (150 mg daily) lowered plasma TG in healthy obese men (37).

It was previously reported by Victor and colleagues that changes in the mitochondrial membrane potentially resulted in the activation of kappa B nuclear factor ( a pro-inflammatory transcription factor) that induces reactive oxygen species (ROS), TNF-α, and endothelial dysfunction in patients with PCOS (38). Although, TNF-α could increase the inflammatory factors such as IL-1, IL-6, and inducible nitric oxide synthase, resveratrol might play an essential role in inhibiting the expression of inflammatory factors (39). A number of studies showed that resveratrol could decrease TNF-α concentration and improve SOD and glutathione peroxidase 1 levels, in addition to disabling superoxide and hydrogen peroxide which is in correlation with the findings of the present study (40, 41). A number of studies suggested that Berberine exerts its anti-inflammatory effects by inhibiting cyclooxygenase-2, which is an enzyme that is responsible for inflammation, as well as nuclear factor kappa-light-chain-enhancer of activated B cells. In this way, it directly scavenges superoxide-free radicals in the system, increases the expression of sirtuin 1, and decreases oxidative stress by reducing expression of nicotinamide and adenine dinucleotide phosphate oxidase which are critical sources for ROS production in cells. In line with the present research, the review study performed by Imenshahidi and co-workers showed that barberry and resveratrol might suppress TNF-α, IL-1, nitric oxide (NO), and MDA through the aforementioned mechanism (9, 19, 42, 43). According to this mechanism, resveratrol and barberry increase SOD and TAC levels. As a result, the simultaneous consumption of barberry and resveratrol increased the antioxidant activity and significantly reduced the inflammation in the PCOS group compared with the metformin group at TAC level. In other words, the simultaneous consumption of barberry and resveratrol led to more desirable results possibly due to their synergistic effects.

The high prevalence of abnormal blood sugar levels in PCOS patients indicates the presence of deficiencies in insulin secretion as well as its function. One study found that androgens could cause insulin resistance and change insulin action in the target tissues in PCOS patients, which may eventually increase visceral adiposity and reduce the secretion of adiponectin, which is the major insulin-sensitizing adipokine (44).

According to the studies, resveratrol and barberry might reduce insulin resistance by regulating insulin signaling pathway through increasing protein kinase B (PKB) expression. This is following the increase in the activity of peroxisome proliferator-activated receptor-gamma (PPAR-γ) and expression of glucose transporter type 4, sirtuin 1, and enhancing glucose uptake in the absence of insulin. In addition, the inhibition of insulin secretion from new island cells, reducing intestinal glucose absorption by inhibiting α-glucosidase activity, and stimulating healthy pancreatic beta cells are other results of this pathway. A study has discovered that berberine reduces insulin resistance in metabolic syndrome (45-47).

The results of this study revealed that the administration of barberry extract and resveratrol in adult female rats with PCOs can lead to significant changes in their insulin resistance.

However, there were no significant changes in the serum glucose levels. This might be a result of low antioxidant dosages and short study duration.

## 5. Conclusion

In conclusion, it can be stated that the combination of Resveratrol and *B. integerrima* (as natural products) can decrease biochemical factors related to the pathogenesis of PCOS. They have the promising antioxidant capacity and anti-inflammatory activities that might ameliorate the complications, and they might be able to regenerate the ovarian morphology to the normal state. Future studies in larger groups, as well as randomized clinical trials on resveratrol and barberry, will help researchers in introducing new treatments for PCOS patients.

##  Conflicts of Interest

None declared.
